# Multi-Analytical Approach to Study Fresh-Cut Apples Vacuum Impregnated with Different Solutions

**DOI:** 10.3390/foods11030488

**Published:** 2022-02-08

**Authors:** Silvia Tappi, Elena Velickova, Cinzia Mannozzi, Urszula Tylewicz, Luca Laghi, Pietro Rocculi

**Affiliations:** 1 Department of Agricultural and Food Sciences, Alma Mater Studiorum—University of Bologna, Piazza Goidanich, 60, 47521 Cesena, Italy; silvia.tappi2@unibo.it (S.T.); l.laghi@unibo.it (L.L.); pietro.rocculi3@unibo.it (P.R.); 2 Interdepartmental Centre for Agri-Food Industrial Research, Alma Mater Studiorum—University of Bologna, Via Quinto Bucci 336, 47521 Cesena, Italy; 3 Department of Food Technology and Biotechnology, Faculty of Technology and Metallurgy, University SS Cyril and Methodius, 1000 Skopje, North Macedonia; 4 Department of Agricultural, Food and Environmental Sciences, Università Politecnica delle Marche, Via Brecce Bianche 10, 60131 Ancona, Italy; c.mannozzi@staff.univpm.it

**Keywords:** minimal processing, isothermal calorimetry, TD-NMR, vacuum impregnation, apples

## Abstract

The aim of this study was to evaluate the effect of different solutions for vacuum impregnation (VI) of fresh-cut (FC) apples through an innovative multi-analytical approach. In particular, the individual and synergistic effects of ascorbic acid and calcium lactate on the preservation of freshness of FC apples was assessed through color, texture, microscopy, isothermal calorimetry, and time-domain nuclear magnetic resonance (TD-NMR) evaluations. The analysis was performed immediately after VI and after 24 h of refrigerated storage. The obtained results showed a good preservation of color and higher firmness in the impregnated samples. Concerning the metabolic heat production, a decrease following the VI treatment was observed, especially when the combined solution was used for the impregnation. The TD-NMR studies showed higher changes in terms of signal intensity and transversal relaxation time T2 after 24 h of storage, evidencing the meta-stability of the plant material for its dynamic tissue nature, and the dewatering/impregnation processes evolution until the achievement of dynamic equilibrium.

## 1. Introduction

Modern lifestyles have led to an increase in consumer interest in healthy food snacks that are safer, convenient, and easy to preserve and use. These requirements have increased the demand of fresh (F), fresh-cut (FC), or minimally processed (MP) food devoid of synthetic chemical preservatives [1,2,3,4]. Obtaining the required food quality and safety of fresh produce is very challenging for the food industry because there are only a few processing technologies able to maintain the product freshness. The most important objectives of fresh-cut food processing companies are control of enzymatic browning and the microbiological safety of fresh-cut produce [5]. However, according to consumers, the distinctive quality of fruits and vegetables is usually described by four different attributes: color and appearance; flavor; and texture and nutritional value [6].

MP is emerging as an alternative technique that produces fresh-like, nutritious, healthy, and easy-to-use foods. Fresh-cut apples are especially convenient for serving among catering services, salad-bars, schools, and company cafeterias. Nevertheless, MP products are characterized by high perishability, because minimal processing operations (e.g., peeling, cutting, dicing) result in loss of cellular integrity that in turn leads to quality deterioration expressed with intensive water loss, softening, microbial contamination, increased respiration, and cut-surface browning [7,8,9].

Recent developments in the shelf-life extension of fresh-cut fruits include physical (modified-atmosphere packaging, pressurized inert gases, electron beam irradiation, pulsed light, ultraviolet light, and cold plasma), chemical (sulfites, acidic electrolyzed water, washing and sanitizing solutions, chlorine, organic acids, hydrogen peroxide, calcium based dips, ozone, and cold gas plasma), and biopreservation (bacteriophages, bacteriocins, and bioprotective microorganisms) technologies. Among these techniques, modified- atmosphere packaging and washing and sanitizing methods are the most industrially used due to their simple procedure [9,10].

Different types of chemical washes are still the most successful methods, but they often raise health concerns regarding residues. Traditionally, sulfites are used to prevent browning; but their use has been banned by the FDA due to concerns over allergic reactions in some individuals [5]. Nowadays, the most widely used alternative to sulfite is ascorbic acid (AA) dipping solution. AA is an antioxidant that is generally recognized as safe (GRAS) (U.S. Food and Drug Administration,1986; 2011). Concerning the calcium-based dips, growing attention has been given to calcium lactate due to its texture firming properties [11,12,13,14,15].

Vacuum impregnation (VI) is a minimal processing technology based on the hydrodynamic mechanism that enables gas replacement in porous food structure, with an aqueous solution resulting in specific formulations of food in short treatments [16]. Vacuum impregnation with hypertonic solutions has been heavily studied, but recently there has been an increased research in vacuum impregnation with isotonic solutions, due to the better preservation of sensory characteristics which makes the MP fruits more appealing to consumers [17,18]. The use of various solutes has been investigated for the production of nutritionally fortified fruit products [19,20] or with the aim of increasing fruit stability [21,22,23]. However, the influence of the solute addition to the impregnating solution on some important qualitative and metabolic parameters of the fresh tissues is not fully known.

In addition, MP products are metabolically active tissues, and their metabolism is strictly correlated to their shelf-life. Isothermal calorimetry, generally coupled with respiration rate measurements, has been previously demonstrated to be a useful tool for assessing the effect of various treatments on MP tissue metabolism and shelf-life [24].

Time-domain nuclear magnetic resonance (TD-NMR) allows the determination of water state and properties (content and mobility) in different cell compartments of different food matrixes by evaluating proton relaxation times [25]. This technique has given valuable information on the effect of different pre-treatments of plant tissues [13,26,27,28].

Different pre-treatments and solutes can impact cell viability in the tissue, influencing the product quality and shelf-life. Microscopic techniques can be used to verify cell viability after treatment by using vital dyes.

For micro- and ultrastructural microscopy, calorimetry and NMR have been successfully employed in investigations of plant foods subjected to mild processing conditions [24,26,29,30].

In this research, a multi-analytical approach that combines several techniques such as colorimetry, empirical rheology, microscopy, isothermal calorimetry, and TD-NMR was assessed to study the effect of different solutes for the vacuum impregnation of fresh-cut apples. The single and synergistic effects of ascorbic acid and calcium lactate, dissolved in isotonic sucrose solution, on the preservation of freshness of the impregnated apples, immediately after treatment and after 24 h of storage in controlled conditions were evaluated.

## 2. Materials and Methods

### 2.1. Raw Materials

Apples (*Malus domestica* Borkh) of the Cripps Pink variety were bought at the local market and stored at 5 ± 1 °C for two weeks, during which all the experiments were performed. Apples were characterized by a soluble solid content of 13.2 ± 0.3 g/100 g fw, dry matter of 9.8 ± 0.7 g/100 g fw, and porosity of 24.09 ± 1.24% (determined according to Velickova et al. (2014) [2]. Cylindrical samples (8 mm diameter, 40 mm length) were cut with a manual cork-borer and a manual cutter designed for the purpose. Commercial sucrose (refined sugar, Eridania, Italy), l-ascorbic acid (Shandong Luwei Pharmaceutical Co., Zibo, China), and calcium lactate (calcium-l-lactate 5-hydrate powder, PURACAL^®^ PP Food, Corbion PURAC, Amsterdam, The Netherlands) were used for the experiments.

### 2.2. Solutions for Impregnation

Impregnating solutions were prepared with sucrose at isotonic concentration (as assessed by the soluble solid content (°Brix) value) compared to apples. Isotonic impregnating solutions were selected to avoid any osmotic phenomenon during treatment. Moreover, 1 g/100 g of ascorbic acid (AA) and 2 g/100 g of calcium lactate (CaLac) alone or in combination were added into the solution. All solutions were analyzed for pH, soluble solid content, density, and viscosity.

### 2.3. Vacuum Impregnation Procedure

An automatic vacuum controller system (AVCS, S.I.A., Bologna, Italy) connected to a closed chamber and a vacuum pump was used for the vacuum impregnation. The impregnation was carried out for 14 min at ambient temperature. An average of 55 ± 1 g of apples (50 cylinders) was immersed in the solutions in a ratio of 1:10 (*w/v*). The impregnation time comprised of a gradual increase of the vacuum for 2 min (100–80 kPa for 30 s, 80–60 kPa for 30 s, 60–40 kPa for 30 s, and 40–20 kPa for 30 s), a holding time of 5 min at 20 kPa and gradual restoring of atmospheric pressure for 2 min. A relaxation time of 5 min was applied after impregnation. Afterwards, samples were removed from the solution, blotted with absorbing paper, and weighed. Three independent impregnations were carried out for each treatment. After impregnation the weight gain (WG) was calculated using the following equation, according to Tylewicz et al. (2012) [31]:

WG = 100 × (m − m_0_)/m_0_ (1)

where m is the mass of the impregnated sample and m_0_ is the initial mass.

Impregnated apple samples were then randomly divided for further analysis immediately after the treatment (non-equilibrated samples) and after 24 h of storage (equilibrated samples). Fresh and vacuum-impregnated (VI) samples were stored in polypropylene cups with lids overnight at 10 °C. Table 1 presents all samples taken into consideration and their abbreviations.

### 2.4. Analytical Methods

#### 2.4.1. Physico-Chemical Parameters

The moisture content of all samples was determined gravimetrically by drying at 70 °C until a constant weight was obtained. Ten cylinders from fresh and impregnated apples were squeezed and filtered, and the obtained juice was used for determination of soluble solid content and pH. Soluble solids were measured at 20 °C with a digital refractometer (PR1, Atago, Japan) previously calibrated with distilled water. pH of the impregnating solutions and fruit samples was measured with a pH-meter (Crison, Barcelona, Spain). The density of the solution was evaluated by weighting 1 mL of solution. The viscosity was determined by a vibrational viscometer (Viscosilite 700 Hydramotion), calibrated with distilled water (viscosity = 1cP). All determinations were carried out at least in triplicate.

#### 2.4.2. Color

To measure the color of apple samples, a spectrophotocolorimeter (ColorFlexTM A60-1010-615, HUNTERLAB, Reston, VA, USA) was used. The instrument was calibrated before use and set with D65 illuminant and the 10° standard observer. The average of at least ten measurements was calculated. The parameters L* (lightness), a* (greenness), and b* (yellowness) were obtained and fitted in several equations, to calculate some specific color changes, such as browning index (*BI*), using Equation (2).

*BI* represents the entity of browning in the apple flesh due to enzyme activity, which can cause deleterious changes in the appearance and organoleptic properties of the food, and therefore it is considered as an important parameter associated with the browning of the samples [32].

(2) $$BI=\frac{100}{0.17}\left(\frac{a*+1.75L*}{5.645L*+a*-3.012b*}-0.31\right)$$

#### 2.4.3. Texture

Texture measurements were conducted by means of a texture analyzer (TA-HDi500, Stable Micro Systems, Surrey, England) equipped with a 50 N load cell. To determine the texture of apple samples, a penetration test was applied. Apple cylinders were placed on the platform and a 2 mm cylinder probe was used to puncture them in the center. The probe moved with cross-head speed of 1 mm/s and trigger force 1 g until a maximum deformation of 90%. The pre- and post-test speed was set at 10 mm/s. The acquired curves (force (N) vs. time (s)) were analyzed and hardness—the first peak force value (N), crunchiness—the linear distance (mm) and gradient (N/s)—as index of compressibility, were extracted.

The effect of the impreganting solution on the vacuum-impregnated apple tissue was calculated as follows:

Impregnating effect = ln Fs/Fc (3)

where Fs is the texture parameter value of the impregnated fruit and Fc is the texture parameter value of the fresh/control fruit (immediately after impregnation—time 0).

#### 2.4.4. Metabolic Heat Production

Fresh cylindrical samples and VI samples were placed in 20 mL glass ampoules and sealed with a Teflon-coated rubber seal and an aluminum crimp cap. Three replicates for each treatment were performed. The rate of heat production was continuously measured in a TAM air isothermal calorimeter (Thermometric AB, Järfälla, Sweden) that is described in detail by Wadsö and Gómez Galindo (2009) [33]. As reference material, water was chosen, and its amount was calculated according to Panarese et al. (2012) [26]. The analysis was performed at 10 °C for 16 h. The first 4 h of analysis were discarded because of the instability of the signal due to the sample loading and conditioning.

#### 2.4.5. Respiration Rate

Immediately after the ampoules were discharged from the calorimeters, the O_2_ and CO_2_ percentages were measured in the ampoule headspaces by a check-point gas analyzer O_2_/CO_2_ (MFA III S/L, Witt-Gasetechnik, Witten, Germany). The apparatus has a paramagnetic sensor for O_2_ and a mini-IR spectrophotometer for CO_2_ detection. The instrument was calibrated with O_2_ and CO_2_ air percentages. The respiration rate was calculated as mol of consumed O_2_ (*RRO*_2_*)* or produced CO_2_ (*RRCO*_2_) h^−1^g^−1^, according to the following equations:

(4) $$RR_{O2}=\frac{V_{head}\cdot \frac{\left(20.8-\%O_{2,head}\right)\cdot P}{100}}{t\cdot m\cdot R\cdot T}$$

(5) $$RR_{CO2}=\frac{V_{head}\cdot \frac{\left(\%CO_{2,head}\right)\cdot P}{100}}{t\cdot m\cdot R\cdot T}$$

where *V* head represents the ampoule headspace volume (dm^3^), *%O*_2_, *head* and *%CO*_2_, *head* refer to molar gases percentages in the ampoule headspace at time t (h), m is the sample mass (g); *R* is the gas constant (8.314472 dm^3^ kPa K^−1^ mol^−1^), *P* is the pressure (101.325 kPa), and *T* is the absolute temperature (283.15 K).

### 2.5. Microscopic Analysis

#### 2.5.1. Fluorescein Diacetate (FDA) Staining

The viability of the cells was evaluated by using fluorescein diacetate (FDA, Sigma-Aldrich, St. Louis, MO, USA, λex = 494 nm, λem = 521 nm), as described by Gómez Galindo, and Toledo and Sjoholm (2005) [34] with some modifications. The apple samples were cut in 2 mm thick slices using sharp razor blades. The discs were incubated for 5 min in an isotonic sucrose solution (13%, *w/w*) containing 10^−4^ mol/L FDA in the darkness at room temperature. Stained sections were rinsed thoroughly in distilled water for 1 min and examined under fluorescent light in a Nikon upright microscope mod. Eclipse Ti-U (Nikon Co, Tokyo, Japan), equipped with a Nikon digital video camera digital sight (DS-Qi1Mc, Nikon Co, Tokyo, Japan) at a magnification of 20×. Undamaged, viable cells could be easily identified by a bright fluorescence.

#### 2.5.2. Methylene Blue Staining

Another set of microscopic analyses was performed using a methylene blue dye, which is a basic dye. The basic dyes combine with those cellular elements that are acidic in nature. Basic stains carry a positive charge, and they are attracted to the oppositely (negatively) charged cells. The presence of negatively charged molecules (such as polyphosphates like DNA and RNA) in the cell causes the staining phenomenon. Therefore, methylene blue binds well to DNA and can be used to stain the nucleic acids. Furthermore, it stains the dead cells and thus differentiates them from the living cells because the dead cells take up the stain more easily than the live cells. Methylene blue, also, indicates the presence or absence of oxygen. The dissolved oxygen oxidizes the methylene blue (which at the start is colorless) turning it blue (when oxidized) [35]. Methylene blue stain was prepared as 0.1 g/100 g aqueous solution. The apple samples were cut in 2 mm thick slices using sharp razor blades and stained with methylene blue for 30 s then rinsed thoroughly with distilled water. Afterwards the samples were observed under the microscope previously reported using a white light at a magnification of ×10, and micrographs were taken.

### 2.6. Time-Domain Nuclear Magnetic Resonance (TD-NMR)

The proton transverse relaxation time (T2) of all samples was measured for six replicates in an LR-NMR (Minispec, Bruker Corporation, Karlsruhe, Germany) operating at 20 MHz and 24 °C, using the Carr–Purcell–Meiboom–Gill (CPMG) pulse sequence. Fresh and VI samples with an 8 mm initial diameter were cut (approximately 10 mm height) to not exceed the active region of the radio frequency coil, and placed inside the 10 mm outer diameter NMR tubes. Each measurement consisted of 16 k echoes, with each train of echoes separated by a recycle delay of 5 s. The specified instrumental parameters avoided heating the samples and allowed the measurement of the protons with a T2 between 1 and 2000 ms. The acquired CPMG curves were further normalized and analyzed with the UPEN (uniform penalty inversion of multi-exponential decay data) algorithm as described by Mauro et al. (2016) [13].

### 2.7. Statistical Analysis

Descriptive statistics and one-way analysis of variance (ANOVA) were performed on the instrumental parameters to evaluate significant differences among the samples at 95% confidence interval according to Tukey’s test using Minitab 15 statistical software.

## 3. Results and Discussion

### 3.1. Physico-Chemical Parameters

Physico-chemical parameters of the impregnating solutions, prepared as described in Table 1, are given in Table 2. The pH value of the isotonic sucrose solution, used as a control, was 7.2. The different combinations of calcium lactate (CaLac) and ascorbic acid (AA) lowered the pH of impregnating solutions. The lowest pH of about 2.5 was measured for the AA solution, while the CaLac one showed a pH of 6.8 which was the most similar to the control solution. The combination of AA and CaLac was characterized by an intermediate value of 4. Soluble solid content increased with the addition of solutes into the isotonic solution, showing the highest value in the solution containing both agents. The viscosity of the solutions, which could be considered crucial for the entity of impregnation, followed the behavior of soluble solids, ranging from 1.47 to 1.74 cP, typical for Newtonian fluids. A similar trend was observed for the density of the solution, although the differences were not so marked.

The weight gain (WG), pH, dry matter, and soluble solids of all impregnated samples are presented in Table 3. WG was in the range of 20.1 to 23.4%, not showing significant differences among the samples. The recorded values were due to the high porosity of the apple tissue of the Cripps Pink variety (24.09 ± 1.24%), indicating that the low range of increased viscosity solution, even if significant, did not influence the impregnation value, as also observed by Tappi et al. (2016) [36] for melon samples. The dry matter and soluble solid content of the samples seemed not to be affected by the vacuum impregnation (VI) step, nor by the solutions’ different compositions. Only the pH value increased when the solutions containing CaLac was used for the impregnation. After 24 h of equilibrium, all the physico-chemical parameters investigated presented similar values to those seen immediately after the treatment [37].

### 3.2. Color and Texture Parameters

**Figure 1:**
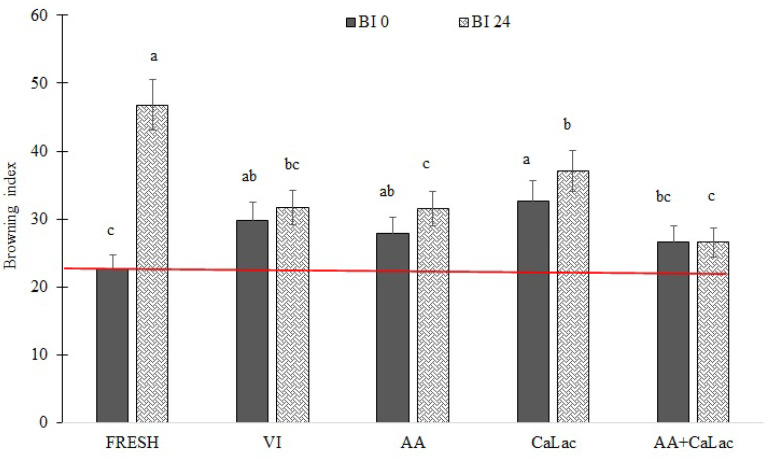
Evaluation of browning index of apple vacuum impregnated with different solutions immediately after impregnation and after 24 h of storage at room temperature. FRESH, control sample; VI, vacuum impregnated with isotonic solution; AA, VI with ascorbic acid; CaLac, VI with calcium lactate; AA+CaLac, VI with both. Bars with different letters indicate significant differences among the samples at the same sampling time.

The effects of the apple impregnation with AA and CaLac on the color parameters L*, a*, and b*, are presented in Table 4, while Figure 1 reflects the browning index (*BI*) of the vacuum-impregnated apple cylinders. The most noticeable changes in the color profile were recorded for the L* parameter. The fresh apples showed L* values of 70.7, while after VI all samples suffered a considerable darkening, with a drop of the L* value in percentages between the 29 and 31% compared to the control sample. Similar behavior has already been reported for impregnated kiwifruit [38] and apple tissues [39,40]. The reason for this change is the alterations of the tissue structural properties due to the application of vacuum and the partial replacement of the gas with a liquid, that promote changes in the refractive index [18,40]. The b* values were also lower after the VI process, but their drop ranged from 10 to 15% and it was not as pronounced as the change in the L* values. The a* values were slightly increased in fresh and sample impregnated with CaLac compared to the fresh one, while the presence of AA allowed similar values to be maintained. Reduced b* and increased a* indicate the occurrence of browning reactions, although only in a mild manner. The browning index was further calculated to better understand the color changes happening in the tissue after impregnation and storage. In agreement with the measured color parameters, a change in the intensity of the brown color of the apples was observed after the impregnation, but it was not very noticeable since the increase of *BI* was between 11 and 25%.

After 24 h storage, the equilibrated fresh samples presented lower L* values and higher a* and b* values and consequently a high *BI* of 46.79, which was 104% higher compared to the fresh fruit. The treated samples resulted in better retention of color in comparison to the fresh equilibrated sample, probably due to the reduced enzyme activity promoted by the reduced presence of oxygen in the tissue [41], and showed only a slight increase of this index when compared with the non-equilibrated samples. In general, the effect of the addition of a singular compound was not observed on the browning inhibition compared to the samples impregnated with only sucrose, while the combined effect of the solutes was significant. According to Chen et al. (1999) [42], the optimal browning inhibition is obtained with solutions containing calcium and ascorbate ions in the ratio of 2:1. Furthermore, Ca-ascorbate in concentrations of 50 and 60 g/L was used to inhibit browning in Braeburn and Ambrosia apples [43,44]. In this research, the sample with Ca-lactate and AA satisfied the 2:1 ratio, exhibiting good browning inhibition.

**Figure 2:**
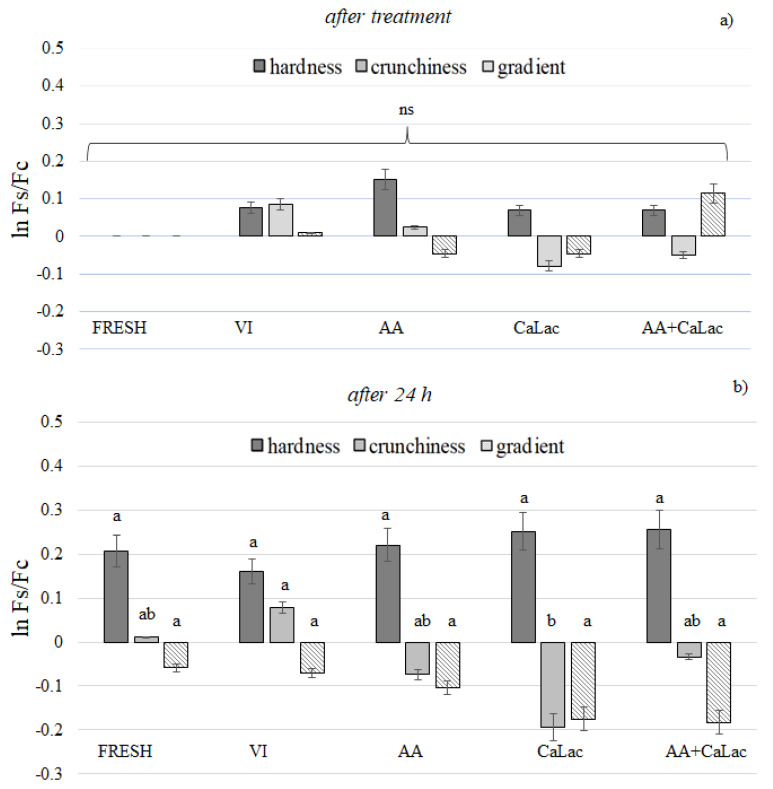
Hardness, crunchiness, and gradient of apple vacuum impregnated with different solutions (**a**) immediately after impregnation and (**b**) after 24 h storage at room temperature. The results are expressed as ln Fs/Fc, where Fs is the texture parameter value of the impregnating fruit and Fc is the texture parameter value of the fresh/control fruit (at time 0). FRESH, control sample; VI, vacuum impregnated with isotonic solution; AA, VI with ascorbic acid; Ca-Lac, VI with calcium lactate; AA+CaLac, VI with both. Bars with different letters indicate significant differences among the samples at same sampling time.

The texture changes of the apple tissue immediately after VI and 24 h storage were also measured. The hardness, crunchiness, and compressibility of the tissue are expressed as the difference in comparison to the fresh tissue immediately after the impregnation (Figure 2a,b). Figure 2 shows that, although differences were not statistically significant because of the high variability of the data, all treatments ended in increased hardness of the apple tissue. It is believed that the endogenous and added calcium can help the retention of the texture because calcium ions cross-link or create bridges between free carboxyl groups of the pectin chains, ending in strengthened cell wall. Furthermore, calcium can also form a complex from the middle lamella polygalacturonic residues and the cell wall in order to improve the structural integrity [45]. Improved textural quality by using calcium lactate was reported for fresh-cut cantaloupe, eggplant, carrot, oyster mushroom, and apple [19,43,46]. However, in the present study, hardness values were similar, irrespective of the solution used for the impregnation.

In accordance with hardness, the crunchiness of the samples did not show significant differences after the treatment due to the high variability of the data. However, after 24 h, the VI sample maintained and even increased its crunchiness, while the addition of Ca-lactate promoted a significant decrease, probably because the increase of the osmotic potential of the impregnating solutions caused a consequent raise in the tissue leaching. The crunchiness of fresh and stored control apple (17.31 ± 2.46 and 17.49 ± 2.34, respectively) was similar to the crunchiness of Pink Lady (16.34 ± 4.46 and 18.87 ± 4.05, respectively) and Fuji apples (17.21 ± 1.99 and 20.91 ± 2.72, respectively) measured by Tappi et al. (2019) [47], at the same storage time (0 and 24 h, respectively). These results confirmed that the crunchiness variation among samples after treatment did not indicate any clear trend, but seemed to be related more to the fruit’s natural variability.

The compressibility of the samples did not show significant changes after VI and storage. Velickova et al. (2018) [48] observed that VI treatment of strawberries combined with pulsed electric field, did not promote any changes in the fruit texture, in terms of the gradient immediately after the treatment. In this study, the changes in the texture of strawberries, which are far more delicate than the apple tissue, were recorded only after a freezing/thawing cycle.

Texture measurement is an empirical imitative analysis, that provide indexes that can be correlated to the sensorial ones. However, to have clear indications about the effect of the treatments, the effect should also be tested also on consumer perception.

### 3.3. Microscopic Analysis

**Figure 3:**
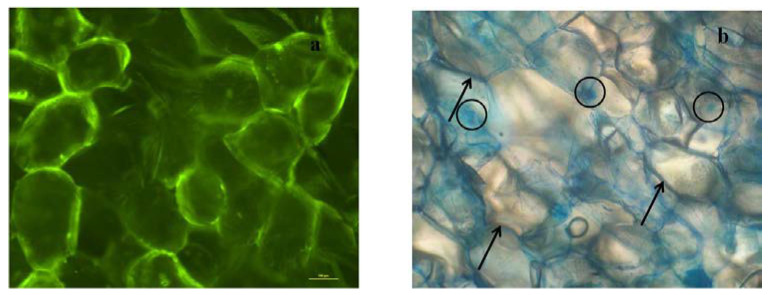
Microphotographs of fresh parenchyma apple tissue stained with (**a**) FDA and (**b**) methylene blue. Arrows point to the cell walls and circles point to the nuclei of the cells.

Figure 3 presents examples of microphotographs of fresh parenchyma apple tissue stained with FDA and methylene blue in order to evaluate the apple tissue cell viability after vacuum impregnation at 24 h storage. FDA is able to passively diffuse through an intact membrane where it is hydrolyzed to a fluorescent compound that cannot move back across the membrane. Hence, a viable cell is characterized by bright green fluorescence, as shown by the fresh tissue (Figure 3a). On the other hand, in living cells, methylene blue binds to the nucleic acids in the cell wall enabling their visualization (Figure 3b, arrows point to the cell walls, circles are drawn around the nucleus of the cells). After cell structure disruption, the dye can easily penetrate inside the cells staining the dead tissues.

**Figure 4:**
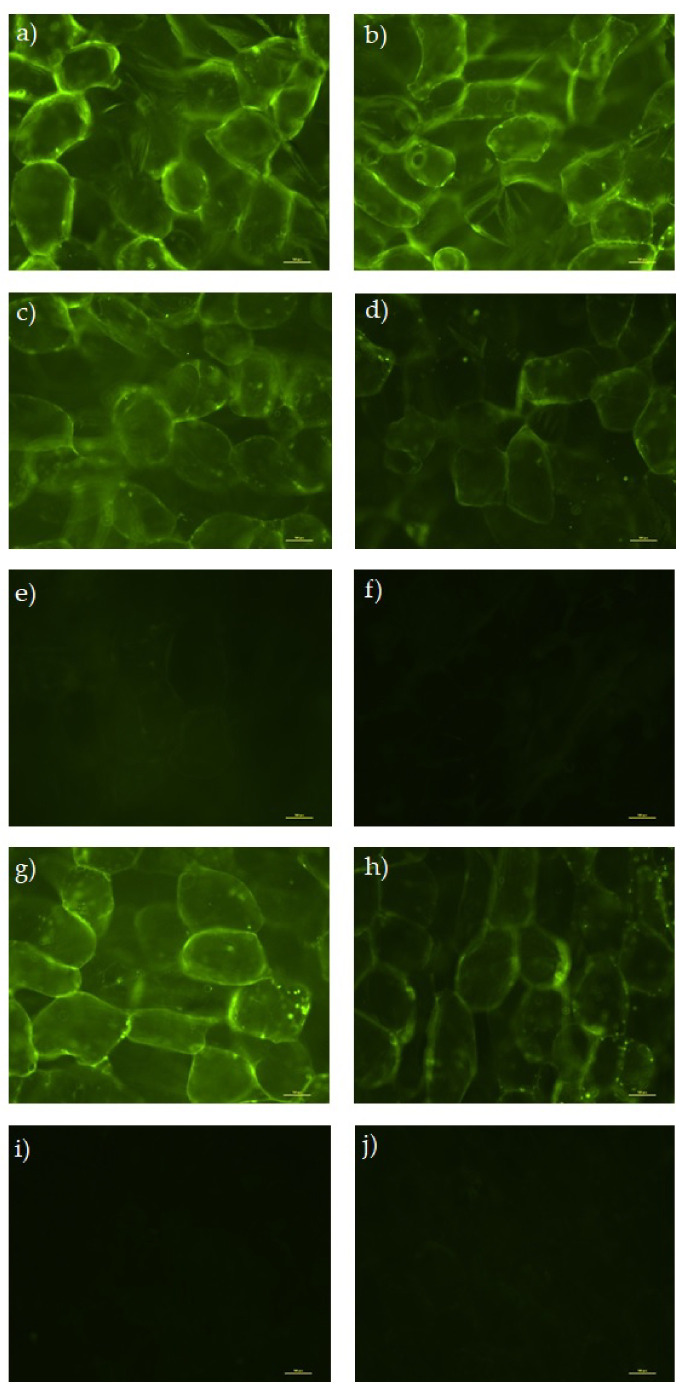
Cell viability of parenchyma apple tissue stained with FDA after vacuum impregnation and 24 h storage. (**a**) Fresh at 0 h, (**b**) fresh at 24 h, (**c**) VI at 0 h, (**d**) VI at 24 h, (**e**) AA at 0 h, (**f**) AA at 24 h, (**g**) CaLac at 0 h, (**h**) CaLac at 24 h, (**i**) AA+CaLac at 0 h, and (**j**) AA+CaLac at 24 h. Fresh, control sample; VI, vacuum impregnated with isotonic solution; AA, VI with ascorbic acid; Ca-Lac, VI with calcium lactate; AA+CaLac, VI with both.

**Figure 5:**
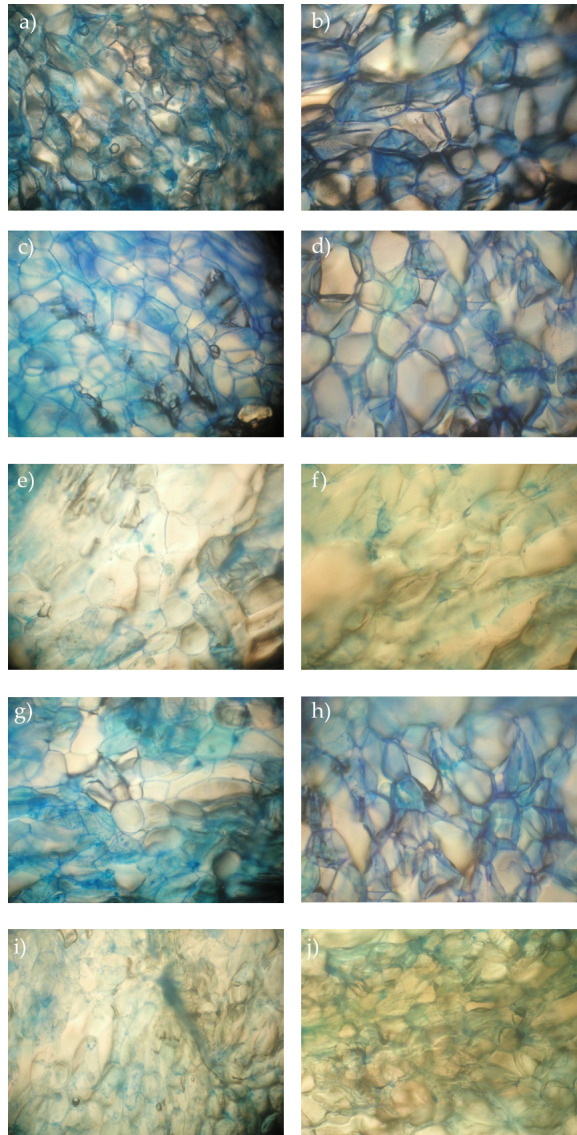
Cell viability of parenchyma apple tissue stained with methylene blue after vacuum impregnation and storage. (**a**) Fresh at 0 h, (**b**) fresh at 24 h, (**c**) VI at 0 h, (**d**) VI at 24 h, (**e**) AA at 0 h, (**f**) AA at 24 h, (**g**) CaLac at 0 h, (**h**) CaLac at 24 h, (**i**) AA+CaLac at 0 h, and (**j**) AA+CaLac at 24 h. Fresh, control sample; VI, vacuum impregnated with isotonic solution; AA, VI with ascorbic acid; Ca-Lac, VI with calcium lactate; AA+CaLac, VI with both.

Microscopic observations of impregnated apple samples stained with FDA and methylene blue are presented in Figure 4 and Figure 5, respectively. The cell viability was well preserved in samples impregnated with isotonic sucrose solutions and solutions with Ca-lactate (bright staining with FDA, Figure 4c,d,g,h and blue staining with methylene blue, Figure 5c,d,g,h). The retention of cell turgor and viability after vacuum impregnation with isotonic sucrose solutions was already reported for apples and spinach [49,50]. Mauro et al. (2016) [13] noticed that concentration of sucrose and Ca-lactate of the up to 20% and 2%, respectively, were able to preserve cellular structure, while increasing over these values caused a deleterious effect. Other researchers observed that the combined treatment of Ca-lactate and warm heating (up to 50 °C) showed more effectiveness in maintaining the turgor of cortex tissue cells in iceberg lettuce and carrots [10,51].

The samples impregnated with AA did not show any cell viability after the impregnation (no fluorescence with FDA, Figure 4e,f,i,j). It seemed that the exogenous AA that penetrated the apple tissue stayed in the intercellular space and was therefore more exposed to oxygen and rapid oxidization leading to cellular death. Moreover, the pH of the solutions (Table 2) can cause a deleterious effect on cellular structure [43,52], similarly to results reported by Qian et al. (2014) [53] and Mauro et al. (2016) [13].

### 3.4. Metabolic Heat Production and Respiration Rate

**Figure 6:**
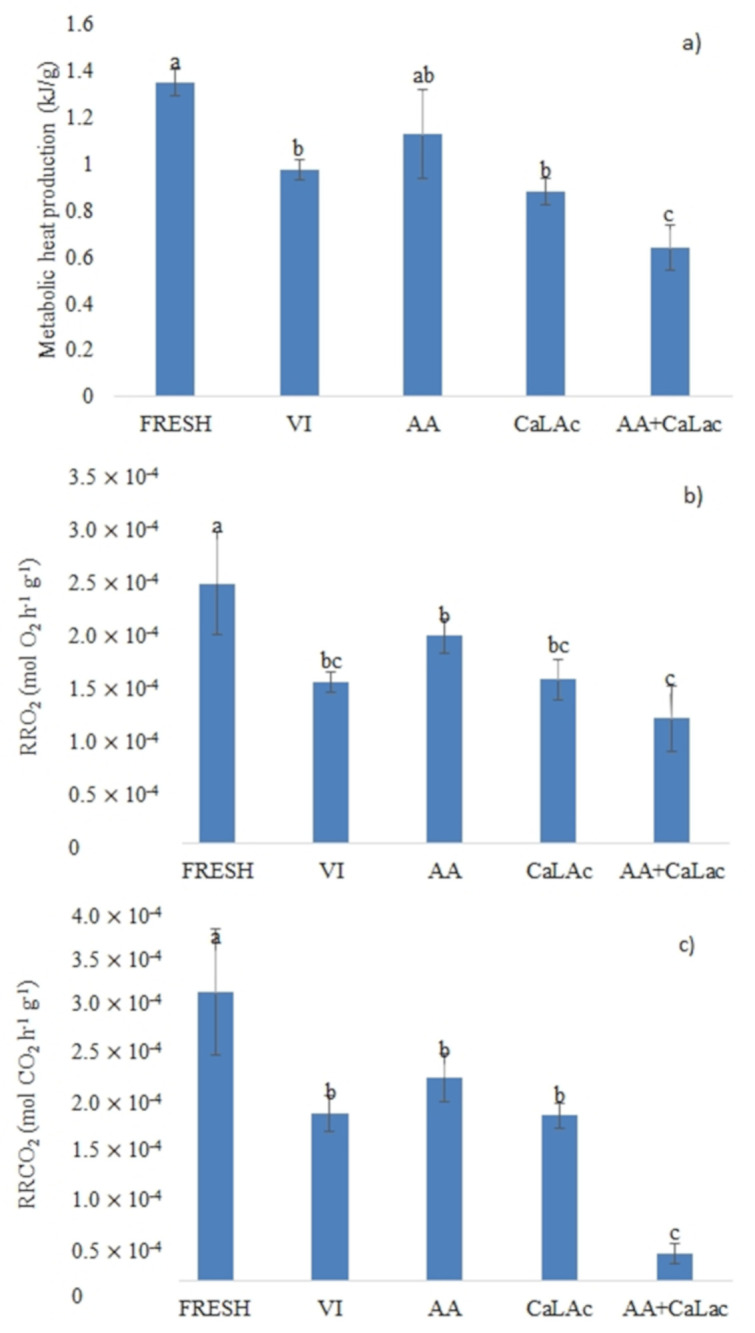
Metabolic heat production measured by isothermal calorimetry (**a**) and respiration rate expressed as O_2_ consumption (**b**), and CO_2_ production (**c**) of fresh and vacuum-impregnated samples. Bars with different letters indicate significant differences among the samples. Fresh, control sample; VI, vacuum impregnated with isotonic solution; AA, VI with ascorbic acid; Ca-Lac, VI with calcium lactate; AA+CaLac, VI with both.

Figure 6 reports the metabolic parameters measured in apple samples through isothermal calorimetry and head space composition. The production of metabolic heat during 16 h of the fresh sample was 1.35 kJ/g. After VI, a significant reduction in the heat produced was observed. The addition of 1 g/100 g AA caused a slight increase, similar to the values of the fresh samples, while no difference was observed when 2 g/100 g CaLac was added in comparison to the only VI. An interesting effect was observed when the two solutes were used in combination. In fact, a significant reduction of the metabolic heat produced was found. Similar behaviour was observed for the respiration rates measured in terms of oxygen consumed and carbon dioxide produced.

Measurements of metabolic heat production by isothermal calorimetry as a consequence of VI have been carried out in various researches by the same group [54,55], mainly on leafy vegetables such as spinach. Results showed a sharp increase of the metabolic activity of the samples when impregnated with isotonic solution of both sucrose and trehalose. This increase was attributed to the metabolization of the sugar by the cells. The results of this research seem to go in the opposite direction. This difference might be due to the different type of tissue and response to the vacuum application and to the presence of solutes. In a previous research [40], we observed that vacuum-impregnated apples showed a lower respiration metabolism during storage which is consistent with a reduced production of gross metabolic heat.

The decrease in respiration rate in terms of oxygen consumption as a consequence of VI was also observed by Igual et al. (2008) [56] on persimmons. However, no differences were observed in CO_2_ production. The authors suggested that this behaviour was due to the lower diffusivity of oxygen in the solution occupying the intercellular spaces of the impregnated sample. This caused an increase of the respiratory quotient indicating the onset of anaerobic respiration of the tissue.

In our research, the decrease of respiration rate was observed for both consumed oxygen and produced CO_2_, with a respiratory quotient (*RRCO_2_/RRO_2_*) that remained quite constant in the range of 1.1–1.2 for all sample apart from AA+CaLac. This result may indicate that the application of VI reduced the respiratory metabolism without changing its pathway, confirming the reduction in metabolic heat production.

However, the sample impregnated with AA+CaLac showed an interesting behavior. While oxygen consumption was not different compared to VI and CaLac samples, the production of CO_2_ was significantly lower. A similar result was previously observed after osmotic dehydration in the presence of both AA and CaLac by Tappi et al. (2017) [14], confirming that the combination of these solutes promotes some unexpected consequences.

In the case of reduced CO_2_ production, the oxygen consumption cannot be derived from respiratory metabolism, but could be explained by the presence of some enzymatic reactions which contribute to an “apparent respiration” [56].

At the moment, it is not possible to put forward a precise explanation of this behaviour; to further clarify the effect of the combination of the solutes, a deeper metabolic evaluation should be carried out, possibly considering the produced metabolites that could give an indication of the metabolic pathways triggered by the treatments.

### 3.5. Time-Domain Nuclear Magnetic Resonance (TD-NMR)

Table 5 shows the relative intensity and the T_2_ and of the proton populations that were observed in the different apple samples. Two proton populations could be systematically identified in each sample, with T_2_ around 1200–1500 and 300 ms. From previous investigations, they were, respectively, ascribed to protons located in the vacuole (Vac) and in the cytoplasm/extracellular space (Cyt/ExS) [13,28]. The relative intensities of Vac and Cyt/ExS signals of fresh apples corresponded to 65 ± 5 and 35 ± 5, respectively. Vacuum impregnation with isotonic sucrose solution did not lead to any significant difference in the proton pool ratios, while 24 h after the treatment a significant release of water from vacuoles to the cytoplasm/extracellular spaces was observed. Hence, even if the sucrose solution was prepared to be isotonic (same water activity value), results may indicate that a small but significant difference in the osmotic potential of the solution and of the cell sap was found.

However, when 2 g/100 g CaLac was present in the solution no significant differences were observed in the intensity of the proton pools, nor after VI, or after 24 h. This discrepancy might indicate that the differences found in fresh apples might be due to the natural variability of apple tissue. However, the changes occurring after 24 h suggest that the plant material is a dynamic tissue and the dewatering/impregnation process evolves in time until the equilibrium is reached [37].

When 1 g/100 g AA was added alone or in combination with 2 g/100 g CaLac into the impregnating solution, a significant shrinkage of the vacuoles was observed, expressed by a higher loss of water from vacuoles. This effect, that was observed straight after VI and confirmed after 24 h storage, can be attributed to the higher osmotic potential of solution containing AA.

Concerning T_2_, immediately after the VI treatment, a significant increase of this value in vacuoles was observed for all samples compared to the fresh one, showing the highest value in samples treated with 2 g/100 g CaLac. This could be due to the difference in the channel selectivity of the plasma and vacuole membranes for several original cell substances, as explained by Mauro et al. (2016) [13]. In the latest samples, this behaviour was also confirmed 24 h after the treatment, while all other samples presented a value similar to the untreated ones.

Concerning the Cyt/ExS, an increase of T_2_ in samples treated with 2 g/100 g CaLac was observed, while samples treated with 1 g/100 g AA alone or in combination presented a decrease of this value, which was also confirmed 24 h after the treatment. A lower T_2_ value for samples osmotically dehydrated in presence of AA was observed by Mauro et al. (2016) [13] compared to sample treated with only sucrose. Dellarosa et al. (2018) [57] observed a general increase of the T_2_ values of vacuum-impregnated samples, which was attributed to the complete replacement of air with the external solution, maintaining the membrane integrity [58].

## 4. Conclusions

The results showed that the different composition of the impregnating solution had a significant effect on the considered quality and metabolic parameters. All impregnated samples resulted in a better retention of color in comparison to the fresh samples and showed only a slight increase of the browning index when compared with the non-equilibrated samples. In general, the impregnated samples showed a higher firmness in comparison to the fresh samples, regardless the treatment applied. Metabolic heat production generally decreased following the VI treatment, but respiration rate measurements confirmed that the aerobic metabolism was maintained.

However, when the combined solution was used for the impregnation, it promoted unexpected results, that although already observed in previous experiments remain difficult to explain and should be further clarified. On the other hand, the microscopic observation showed a loss of viability when ascorbic acid was added to the impregnated solution that was paralleled by a significant shrinkage of vacuoles observed by relaxometry studies. The obtained data can give useful insight to the effects of CaLac and AA applied alone and in combination on the fresh-like characteristics of apples, helping to develop suitable minimal processing methods to maximize the shelf-life and quality of these products. However, the discrepancies observed by the different analytical techniques, after the treatment and during the further equilibration period, confirm the high complexity of the metabolic responses of the tissue that need further investigation.

## Figures and Tables

**Table 1:** Abbreviations for apple samples subjected to the vacuum-impregnation (VI) treatments with different solutions.

Samples	CaLac (g/100 g)	AA (g/100 g)
FRESH	Control sample	
VI	VI sample with isotonic solution	
AA	0	1
CaLac	2	0
AA+CaLac	2	1

**Table 2:** Physico-chemical parameters of the solutions used for vacuum impregnation (VI).

Solution	pH	SSC (Brix°)	Density (kg/m^3^)	Viscosity (cP)
Isotonic	7.2 ± 0.1 ^a^	13.10 ± 0.01 ^d^	1.058 ± 0.003 ^b^	1.47 ± 0.01 ^d^
AA	2.5 ± 0.1 ^d^	14.00 ± 0.01 ^c^	1.059 ± 0.006 ^b^	1.52 ± 0.01 ^c^
CaLac	6.8 ± 0.1 ^b^	14.50 ± 0.01 ^b^	1.067 ± 0.002 ^ab^	1.62 ± 0.02 ^b^
AA+CaLac	4.0 ± 0.1 ^c^	15.60 ± 0.01 ^a^	1.074 ± 0.004 ^a^	1.74 ± 0.01 ^a^

Different letters in a column indicate significant differences among the samples.

**Table 3:** Physico-chemical parameters of the vacuum-impregnated samples immediately after the vacuum impregnation (VI) treatment and after 24 h storage.

Sample	Weight Gain (%)	Dry Matter (g/100 g)	SSC (° Brix)	pH
Immediately after treatment (0 h)				
FRESH	-	9.8 ± 0.7 ^ab^	13.2 ± 0.3 ^a^	3.4 ± 0.01 ^c^
VI	23.4 ± 0.5 ^a^	11.2 ± 0.2 ^a^	13.4 ± 0.1 ^a^	3.3 ± 0.03 ^c^
AA	20.1 ± 3.6 ^a^	7.5 ± 1.6 ^b^	14.2 ± 1.1 ^a^	3.4 ± 0.06 ^c^
CaLac	21.5 ± 5.2 ^a^	9.9 ± 0.6 ^ab^	13.6 ± 0.6 ^a^	3.9 ± 0.02 ^a^
AA+CaLac	20.8 ± 1.3 ^a^	7.9 ± 1.3 ^b^	14.2 ± 1.3 ^a^	3.8 ± 0.06 ^b^
After 24 h				
FRESH		9.9 ± 1.6 ^ab^	13.8 ± 0.5 ^ab^	3.5 ± 0.05 ^c^
VI		11.6 ± 0.3 ^a^	13.1 ± 0.1 ^b^	3.4 ± 0.05 ^c^
AA		8.9 ± 0.8 ^b^	14.2 ± 0.7 ^ab^	3.4 ± 0.04 ^c^
CaLac		9.5 ± 0.3 ^ab^	14.0 ± 0.1 ^ab^	3.9 ± 0.02 ^a^
AA+CaLac		10.7 ± 0.0 ^ab^	14.6 ± 0.3 ^a^	3.8 ± 0.02 ^b^

Different letters in a column indicate significant differences among the samples.

**Table 4:** Color parameters of fresh and vacuum-impregnated samples immediately after the vacuum impregnation (VI) treatment and 24 h storage.

Sample	*L*	*a*	*b*
Immediately after treatment (0 h)			
FRESH	70.7 ± 1.7 ^a^	−1.07 ± 0.15 ^d^	15.1 ± 1.2 ^b^
VI	49.7 ± 1.8 ^cd^	−0.55 ± 0.16 ^c^	13.5 ± 0.7 ^cd^
AA	49.3 ± 2.3 ^cde^	−1.28 ± 0.21 ^de^	13.1 ± 2.2 ^cd^
CaLac	48.4 ± 2.4 ^cdef^	−0.26 ± 0.395 ^b^	13.7 ± 1.6 ^bcd^
AA+CaLac	50.0 ± 1.8 ^c^	−1.17 ± 0.22 ^de^	12.7 ± 0.9 ^d^
After 24 h			
FRESH	65.2 ± 1.1 ^b^	2.9 ± 0.2 ^a^	24.1 ± 1.3 ^a^
VI	47.8 ± 1.6 ^cdef^	0.01 ± 0.11 ^b^	13.1 ± 0.9 ^cd^
AA	46.8 ± 1.4 ^ef^	−0.97 ± 0.25 ^cde^	13.5 ± 1.8 ^cd^
CaLac	47.0 ± 2.6 ^f^	−0.03 ± 0.37 ^b^	14.8 ± 2.1 ^bc^
AA+CaLac	47.1 ± 1.8 ^def^	−1.37 ± 0.150 ^e^	12.1 ± 0.6 ^d^

Different letters in a column indicate significant differences among the samples.

**Table 5:** Peak intensity and relaxation times (T2) of different cell compartments in fresh and vacuum-impregnated samples immediately after the vacuum treatment (VI) treatment and after 24 h storage.

Sample	0 h		24 h	
	Vacuoles	Cytoplasm/ Extracellular Space	Vacuoles	Cytoplasm/ Extracellular Space
Peak intensity (a.u.)				
FRESH	65 ± 5 ^a^	35 ± 4 ^bc^	70 ± 2 ^a^	30 ± 3 ^c^
VI	63 ± 1 ^ab^	37 ± 1 ^ab^	62 ± 2 ^b^	38 ± 2 ^b^
AA	60 ± 2 ^b^	40 ± 2 ^a^	58 ± 1 ^c^	42 ± 1 ^a^
CaLac	66 ± 2 ^a^	33 ± 2 ^c^	68 ± 1 ^a^	32 ± 2 ^c^
AA+CaLac	61 ± 2 ^b^	39 ± 2 ^a^	61 ± 2 ^b^	39 ± 1 ^ab^
T_2_ (ms)				
FRESH	1231 ± 20 ^d^	302 ± 16 ^b^	1284 ± 54 ^b^	309 ± 19 ^b^
VI	1459 ± 25 ^ab^	298 ± 10 ^b^	1279 ± 45 ^b^	320 ± 10 ^b^
AA	1422 ± 47 ^b^	272 ± 16 ^c^	1256 ± 74 ^b^	277 ± 11 ^bc^
CaLac	1514 ± 58 ^a^	369 ± 12 ^a^	1479 ± 31 ^a^	371 ± 9 ^a^
AA+CaLac	1347 ± 48 ^c^	276 ± 9 ^c^	1234 ± 34 ^b^	39 ± 1 ^ab^

Different letters in a column indicate significant differences among the samples.

## Data Availability

Not applicable.
