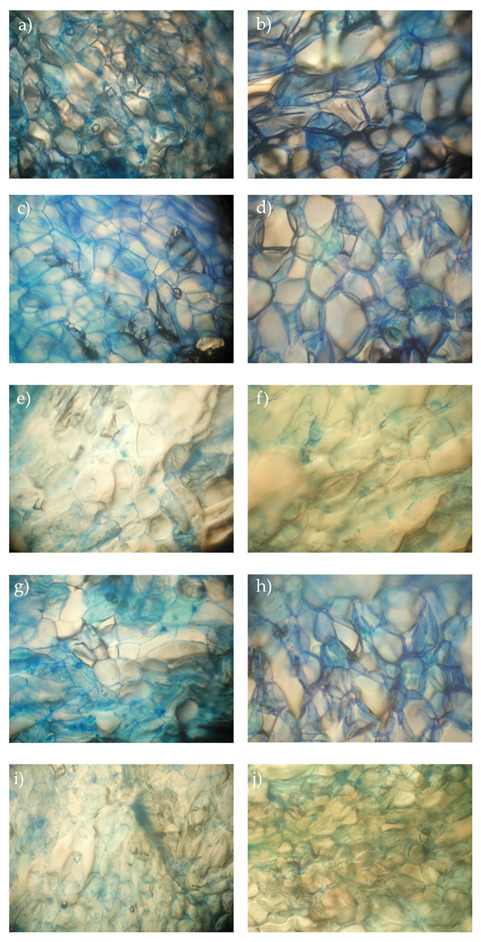# Correction: Tappi et al. Multi-Analytical Approach to Study Fresh-Cut Apples Vacuum Impregnated with Different Solutions. *Foods* 2022, *11*, 488

**DOI:** 10.3390/foods13132136

**Published:** 2024-07-04

**Authors:** Silvia Tappi, Elena Velickova, Cinzia Mannozzi, Urszula Tylewicz, Luca Laghi, Pietro Rocculi

**Affiliations:** 1 Department of Agricultural and Food Sciences, Alma Mater Studiorum—University of Bologna, Piazza Goidanich, 60, 47521 Cesena, Italy; silvia.tappi2@unibo.it (S.T.); l.laghi@unibo.it (L.L.); pietro.rocculi3@unibo.it (P.R.); 2 Interdepartmental Centre for Agri-Food Industrial Research, Alma Mater Studiorum—University of Bologna, Via Quinto Bucci 336, 47521 Cesena, Italy; 3 Department of Food Technology and Biotechnology, Faculty of Technology and Metallurgy, University SS Cyril and Methodius, 1000 Skopje, North Macedonia; 4 Department of Agricultural, Food and Environmental Sciences, Università Politecnica delle Marche, Via Brecce Bianche 10, 60131 Ancona, Italy; c.mannozzi@staff.univpm.it

In the original publication [1], there was a mistake in Figure 5 as published. Figure 5b,d were duplicated due to error. The corrected Figure 5 appears below. The authors state that the scientific conclusions are unaffected. This correction was approved by the Academic Editor. The original publication has also been updated.